# Technologies for Biogas Upgrading to Biomethane: A Review

**DOI:** 10.3390/bioengineering6040092

**Published:** 2019-10-02

**Authors:** Amir Izzuddin Adnan, Mei Yin Ong, Saifuddin Nomanbhay, Kit Wayne Chew, Pau Loke Show

**Affiliations:** 1 Institute of Sustainable Energy, Universiti Tenaga Nasional, Kajang 43000, Selangor, Malaysia; izzuddin.amir95@gmail.com (A.I.A.); me089475@hotmail.com (M.Y.O.); 2 Department of Chemical and Environmental Engineering, Faculty of Science and Engineering, University of Nottingham Malaysia, Jalan Broga, Semenyih 43500, Selangor, Malaysia; kitwayne.chew@gmail.com (K.W.C.); pauloke.show@nottingham.edu.my (P.L.S.)

**Keywords:** anaerobic digestion, biogas upgrading, biomethane, bio-succinic acid, CO_2_ utilization, feasibility assessment

## Abstract

The environmental impacts and high long-term costs of poor waste disposal have pushed the industry to realize the potential of turning this problem into an economic and sustainable initiative. Anaerobic digestion and the production of biogas can provide an efficient means of meeting several objectives concerning energy, environmental, and waste management policy. Biogas contains methane (60%) and carbon dioxide (40%) as its principal constituent. Excluding methane, other gasses contained in biogas are considered as contaminants. Removal of these impurities, especially carbon dioxide, will increase the biogas quality for further use. Integrating biological processes into the bio-refinery that effectively consume carbon dioxide will become increasingly important. Such process integration could significantly improve the sustainability of the overall bio-refinery process. The biogas upgrading by utilization of carbon dioxide rather than removal of it is a suitable strategy in this direction. The present work is a critical review that summarizes state-of-the-art technologies for biogas upgrading with particular attention to the emerging biological methanation processes. It also discusses the future perspectives for overcoming the challenges associated with upgradation. While biogas offers a good substitution for fossil fuels, it still not a perfect solution for global greenhouse gas emissions and further research still needs to be conducted.

## 1. Introduction

In the last decades, fossil fuels have been utilized at a high rate as the main energy source for the industrial process as well as daily usage. The result is the increasing crisis of global energy and environmental problems. It has been predicted that the global consumption of energy will increase nearly threefold in the next thirty years [1]. Massive carbon dioxide (CO_2_) emission during fossil fuel combustion has raised the concern on energy sustainability and environmental protection issues. The rate of CO_2_ that is presently being released at a global scale is more than 1000 kg/s, although it is the imbalance between emissions and sinks that is responsible for the increasing CO_2_ concentration in the atmosphere [2]. The reductions of CO_2_ emission into the atmosphere can only be achieved by either reducing the CO_2_ emissions from the sources or increasing the usage of CO_2_. A wide-ranging research plan is needed to develop a variety of carbon utilization technologies suitable for utilizing the abundance of carbon waste in the atmosphere, integrating enabling technologies and resources, and producing a wide range of carbon-based products. Therefore, extensive research needs to be conducted to address the knowledge gaps throughout the carbon utilization landscape in order to reduce greenhouse gas emissions (GHG) while generating economic value. The conversion of CO_2_ into added-value chemicals and fuels is considered as one of the great challenges of the 21st century.

To achieve sustainable development, energy resources with low environmental impact should be utilized. Besides petroleum, biomass is the largest source of carbon-rich material available on Earth [3]. Biorefineries represent tremendous potential for the efficient utilization of renewable resources. A biorefinery can be described as a facility that integrates biomass conversion processes and technologies in a sustainable and efficient way to produce a variety of marketable products (food, feed, chemicals, and materials) and energy (biofuels, power, and/or heat) from biomass. Biogas is a well-established renewable energy source for combined heat and power (CHP) generation. Biogas production is a treatment technology that generates renewable energy and recycles organic waste into a digested biomass, which can be used as fertilizer and soil amendment. Biogas is considered a renewable energy source due to the fact that the organic waste has consumed carbon dioxide in the photosynthesis process, and as such can be described as carbon-neutral [4]. The amount of wastes and residues generated has led to the demand for technologies and processes that can help to reduce these residues, which can help achieve the ambitious objective of “zero-waste” targets (or, at least, waste minimization) while obtaining valuable commodities, including renewable-based methane-rich product gas streams. In these regards, waste management technologies based on the anaerobic digestion of different residual streams, such as municipal solid wastes in landfills, agriculture crops, and urban wastewaters that allow the production of biogas, have played a significant role in the last decades. To date, efforts have been made to improve the methane (CH_4_) yield during anaerobic digestion. Feedstock selection, process design and operation, digestion enhancement, and co-digestion with multiple substrates have been extensively studied, and several reviews are available [5,6,7,8,9].

Commercial biogas production has increased since it can be used as fuel or energy production while contributes to a lower GHG concentration when it is collected in a closed process and not emitted to the atmosphere. Depending on the nature of the substrate and pH of the reactor, biogas produced consists of CH_4_ in a range of 50–70% and CO_2_ at a concentration of 30–50%, with the addition of minor components such as hydrogen sulfide (H_2_S), nitrogen (N_2_), oxygen (O_2_), siloxanes, volatile organic compounds (VOCs), carbon monoxide (CO), and ammonia (NH_3_). It is estimated that biogas usage in the world will be doubled in the coming years, increasing from 14.5 GW in 2012 to 29.5 GW in 2022 [10,11]. Apart from CH_4_, the remaining components in biogas are undesirable and considered as impurities. Basically, there are two steps involved in biogas treatment, cleaning (removal of minor unwanted components of biogas), and upgrading (removal of CO_2_ content) [10,11]. After the processes, the final product is called biomethane which composed of CH_4_ (95–99%) and CO_2_ (1–5%), with no trace of H_2_S. Biogas cleaning is usually considered the first step for biogas applications and is an energy-demanding process. The second treatment is called “biogas upgrading” and aims to increase the low calorific value of the biogas, and thus, to convert it to a higher fuel standard [12]. Nowadays, there are different treatments targeted at removing the undesired compounds from the biogas, thus expanding its range of applications. High CH_4_ purity biogas has the same properties as natural gas, especially in terms of heating value, therefore, this clean biogas is qualified to be injected into a natural gas grid [13]. An early notable review report on biogas upgrading was published in 2009, providing a complete view on the situation of biogas upgrading at that time, however, the topic on CO_2_ removal was only briefly discussed [14]. More review reports on biogas purification and upgrading had appeared recently. The first of them was by Ryckebosch and others (2011) [15] discussing the state of affairs of different techniques for biogas transformation and their functions, efficiency, and barriers. Next, Bauer et al. (2013) [16] reviewed and compared the commercial technologies on biogas upgrading. In later years, Sun et al. (2015) [12] had come out with a more detailed review on biogas upgrading technology, focusing on biogas purity and impurities, CH_4_ recovery and loss, upgrading efficiency, investment, and operating cost. These were among the many reviews that were conducted on the topic of biogas upgrading involving CO_2_ removal. Therefore, in this review, an attempt is made to present new technologies for biogas upgrading via the utilization and conversion of CO_2_ rather than the removal of CO_2_. The already matured technologies will only be briefly summarized.

## 2. Biogas Upgrading via Carbon Dioxide Removal Technologies

**Figure 1:**
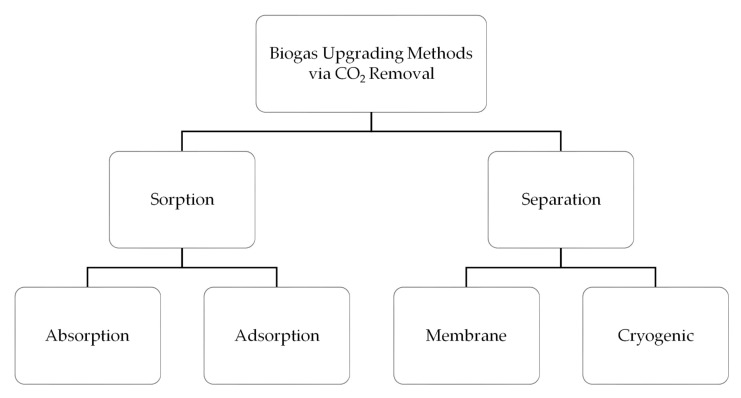
Technologies for biogas upgrading via CO_2_ removal route.

As a means to upgrade biogas to a higher fuel standard, that is, to remove unwanted components such as CO_2_ and H_2_S thus increasing its specific caloric value, several different approaches have been proposed [17,18]. The mature technologies that are today currently applied for biogas upgrading are illustrated in Figure 1. The focus of this section is to summarize the important details regarding current CO_2_ removal technologies rather than going into details on it.

**Figure 2:**
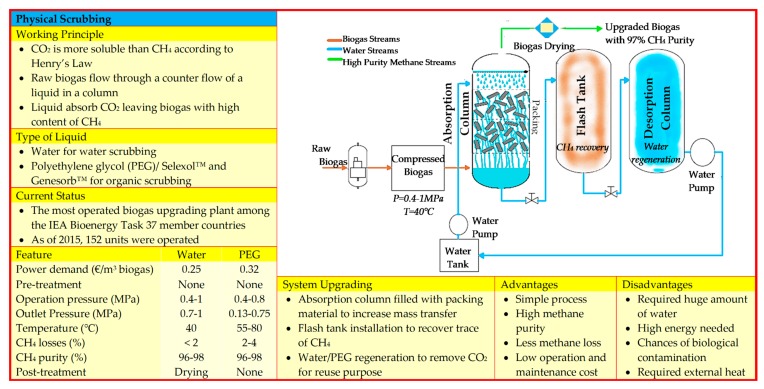
Summary of fundamental knowledge on physical scrubbing technology [15,19,20,21,22,23,24,25,26,27,28].

**Figure 3:**
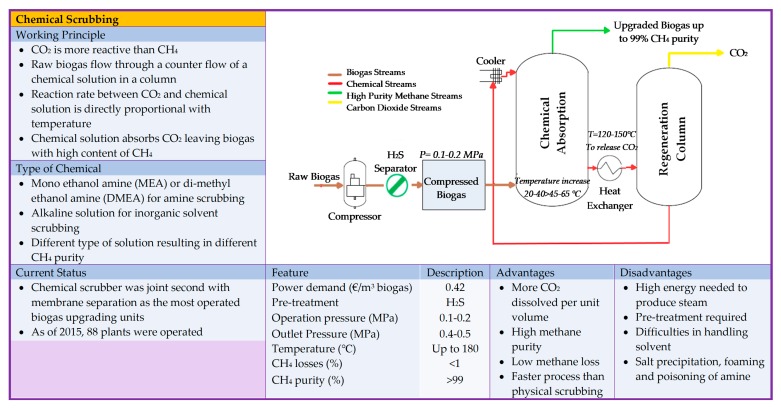
Summary of basic information on chemical scrubbing technology [15,19,20,25,27,28,29,30,31,32].

**Figure 4:**
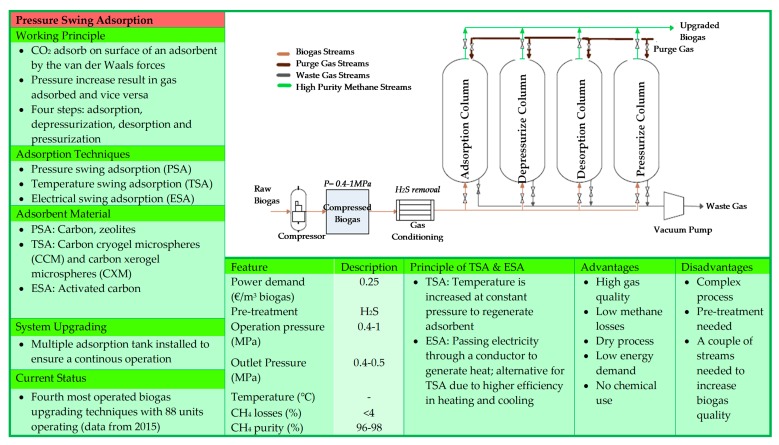
Depicts information on pressure swing adsorption technology [19,20,25,28,33,34,35,36,37].

**Figure 5:**
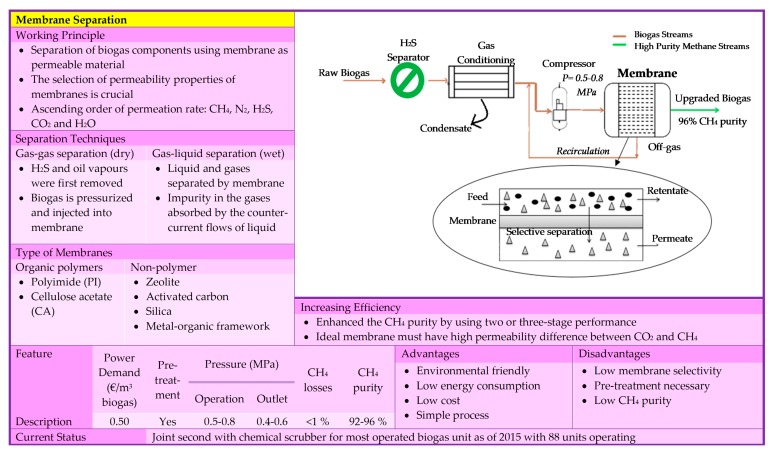
Summary of base knowledge of membrane separation technology [20,21,25,27,28,29,38,39].

**Figure 6:**
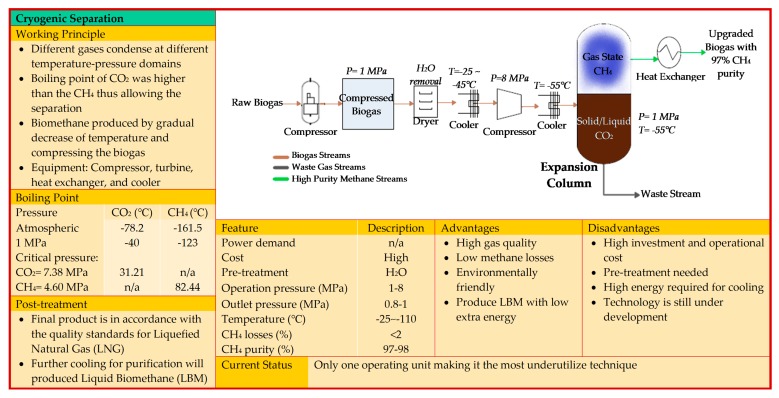
Depicts fundamental knowledge on cryogenic separation [15,19,20,25,28,29,40,41,42,43].

The gas sorption is divided into two categories: physical and chemical scrubbing. Physical scrubbing and chemical scrubbing processes were summarized in Figure 2 and Figure 3 respectively. Next, the adsorption method was usually done in a process called pressure swing adsorption and can be seen as a summarized point in Figure 4. Then, the term separation is applied in membrane technology and cryogenic separation and depicted as in Figure 5 and Figure 6, respectively.

The benefits of biogas to the environment are often discussed as a sustainable source of fuels [44]. However, some biogas components released from biogas upgrading are associated with GHG, especially CO_2_. The direct impacts of excessive CO_2_ emission are global warming, ocean acidification, and carbon fertilization. The released CO_2_ needs to be disposed of. It includes the processes of CO_2_ liquifying and injection into underground aquifers. The drawback of this process is the possibility of CO_2_ leaking and returning to the surface. Furthermore, the cost of CO_2_ disposal is very high and uncertain (among the factors that contribute to cost are the size of the plant and the distance). Thus, a possible solution for this problem is through CO_2_ utilization technology. This technology holds big potential for a new way of upgrading biogas, since the benefits of utilizing CO_2_ could potentially overcome the cost of CO_2_ disposal and reduce the cost of biogas upgrading. The next section of this review will focus on the discussion of various techniques for the utilization of CO_2_ as reported in the literature.

## 3. Biogas Upgrading via Carbon Dioxide Utilization Technologies

In the previous section, biogas was upgraded to enrich the methane content and treated directly as fuel without essential chemical changes. The technologies are always changing, and researchers have developed methods to further explore the value of raw biogas. In recent years, biogas has been used as feedstock in producing chemical material by utilizing the CO_2_ content in the biogas [45]. In addition, this low-grade biogas will benefit society by the production of high-quality products instead of inefficient heat supply that results in higher pollution. This section will discuss the state-of-art of emerging technologies for biogas upgrading through CO_2_ utilization.

### 3.1. Chemical Processes

It is well known that using CO_2_ as a feedstock for the synthesis of commodity chemicals and fuels has the potential to be beneficial for the economy and environment [46]. CO_2_ with the molecular weight of 44.01 and critical density of 468 kg/m^3^ can be in a liquid state at a pressure below 415.8 kPa and in the form of solid under −78 °C. It is a massively produced waste and the main contributor to global warming. Despite the potential, the challenges that arise from the utilization of CO_2_ are the need for large inputs of energy and the strong bonds that are not particularly reactive due to its kinetic and thermodynamic stability. For instance, it is not affected by heat under normal conditions until the temperature reaches about 2000 °C [47]. Consequently, the process of converting CO_2_ requires stoichiometric amounts of energy-intensive reagents that lead to the generation of other waste and increasing GHG footprints. Thus, the main challenge is to develop a new technology that can reduce the use of non-renewable energy and reduce GHG emissions.

Methanation reaction, also called a Sabatier reaction is a reaction between CO_2_ and H_2_ to produce CH_4_ and water (H_2_O). Although the reaction is between CO_2_ and H_2_, there is the potential of using biogas directly as feedstock for CO_2_ methanation as CH_4_ content in the biogas has only a little influence on the reaction at high pressure [48]. The research has found that the methanation of CO_2_ above 0.8 MPa will be ideal to decrease the effect of CH_4_ on the conversion process [49]. CH_4_ is consumed by the consumer widely as a fuel in 2014 (3500 billion cubic meters) [50]. The main source of CH_4_ is natural gas, and occasionally as a result of synthetization. The process of hydrogenation of CO_2_ to CH_4_ using Ni catalyst is explained by Sabatier reaction in Equation (1) [51].

CO_2_ + 4H_2_ → CH_4_ + 2H_2_O   ∆H = −165 kJ/mol (1)

The research in the improvement of catalysts is still developing. Challenges that need to be confronted include the catalysts that can operate at lower temperatures where the reaction more promising and preventing the deactivation of nickel-based catalysts due to sintering and oxidation. Sintering occurs due to the high temperature and water while oxidation is due to the presence of H_2_ [52,53]. The improvement of catalysts and processes that have been recently discovered are simplified in Table 1.

On the other hand, by changing the nature of catalysts to less reactive catalysts result in the production of methanol. In 2015, approximately 70 billion kg of methanol (CH_3_OH) was produced worldwide from the synthetization of syngas (H_2_ + CO_2_) obtained directly from fossil fuels [58,59,60,61]. The mechanism of methanol production, seen in Equation (2), involves a side reaction between CO_2_ and H_2_ to produce CO and H_2_O based on water gas-shift reaction as shown by Equation (3).

CO_2_ + 3H_2_ ↔ CH_3_OH + H_2_O   ∆H_298K_ = −90.70 kJ⁄mol (2)

H_2_ + CO_2_ ↔ CO + H_2_O   ∆H_298K_ = 41.19 kJ⁄mol (3)

The methanol formation here is an exothermic reaction and the molecular weight of molecules with carbon decrease. Thus, there will be an increase in pressure and a decrease in temperature for selectivity. But, as mentioned earlier, CO_2_ is not very reactive and needs a high reaction temperature (>513 K) for CO_2_ conversion to occur. In recent years, a lot of research has been done on the catalysts used for direct hydrogenation of CO_2_ to methanol, and the results have shown that high pressure is needed to achieve high methanol selectivity [58,62,63]. The most suitable catalyst is not yet available in the current industry. Two challenges for catalyst development are the huge amount of water produced by both reactions that inhibit the product and the undesirable reverse water gas–shift reaction that consumes hydrogen, thus results in a decrease in the yield for methanol. Copper-zinc-aluminum oxide catalyst is often used in CO_2_ hydrogenation. The process is run at 5.0–10.0 MPa and 473–523 K. But, the catalyst is not effective again for hydrogenating pure CO_2_ [64]. Significant amounts of research into the direct hydrogenation of CO_2_ to methanol is continuing. Some of the researches are simplified in Table 2.

Another product that can be obtained from the methanation of CO_2_ is carbon monoxide. CO is usually obtained through partial oxidation of hydrocarbons or coal at high temperatures around 800 °C. CO is a valuable feedstock in the synthesis of different commodities such as methanol and other higher-order hydrocarbons. The method of obtaining CO from CO_2_ from the methanation process is the reverse water–gas shift reaction (shown in Equation (3)) as the major by-product [68]. The reaction is endothermic and requires s high temperature (~500 °C). A wide range of heterogeneous catalysts often used are copper-, iron-, or ceria-based systems for the reverse water–gas shift reaction. The problems of these catalysts are poor thermal stability and undesired side product often formed. Due to this thermodynamic constraint, it is unlikely for the research on converting CO_2_ to CO using reverse water–gas shift reaction to advance beyond this stage. Furthermore, there are other potential routes to generate CO from CO_2_ at a significantly more advanced state. To directly reduce CO_2_ to CO and O_2_, the use of electrochemical splitting provides an alternative way. Unfortunately, the subject will not be discussed further in this paper, but information on the process can be obtained here [69,70].

### 3.2. Biological Processes

Biological processes complement chemical options due to its uniqueness of carbon utilization resource requirements and product opportunities. It focuses on the aptitude of microorganisms to convert CO_2_ into useful products. Biological fixation of CO_2_ is a sustainable solution to reduce CO_2_ content in biogas due to its nature which is environmentally-friendly and eliminates the step of captured CO_2_ disposal [71]. One of the biological methods to utilize CO_2_ in biogas relies on the utilization of H_2_ for the conversion of CO_2_ to CH_4_ based on the action of hydrogenotrophic methanogens. The reaction is shown in Equation (4). 

4H_2_ + CO_2_ → CH_4_ + 2H_2_O   ∆G° = −130.7 kJ/mol (4)

The source of H_2_ is the hydrolysis of water. To ensure the method is sustainable, electricity needed in the hydrolysis process came from renewable sources, such as solar and wind. One of the disadvantages of H_2_ was its low volumetric energy density, resulting in storage difficulties [72]. This H_2_ assisted biogas upgrading can occur in a so-called in-situ and ex-situ biological biogas upgrading. Ex-situ upgrading had been discussed in previous sections and includes absorption, adsorption, membrane separation, and cryogenic methods. It requires the CO_2_ to be removed first, thus defeating the purpose of utilizing the CO_2_ in biogas, which is the focus of this topic. Ex-situ upgrading will not be discussed further but the review can be found here [73]. Meanwhile, the process of in-situ upgrading does not require the CO_2_ to be removed first, rather it will be converted into CH_4_ leading to a significant increment in biogas purity [13].

In-situ biological biogas upgrading uses the injection of H_2_ inside a biogas reactor during anaerobic digestion to react with CO_2_, resulting in CH_4_ production by the action of autochthonous methanogenic archaea [13]. This can be operated through two different pathways: hydrogenotrophic methanogenesis and Wood–Ljungdahl [74]. Hydrogenotrophic methanogenesis performs direct conversion of CO_2_ to CH_4_ with the addition of H_2_ as a source of electrons, according to Equation (4). Meanwhile the Wood–Ljungdahl pathway indirectly converts CO_2_ to CH_4_ via two reactions according to Equations (5) and (6).

4H_2_ + 2CO_2_ → CH_3_COOH + 2H_2_O   ∆G° = −104.5 kJ/mol (5)

CH_3_COOH → CH_4_ + CO_2_   ∆G° = −31.0 kJ/mol (6)

**Figure 7:**
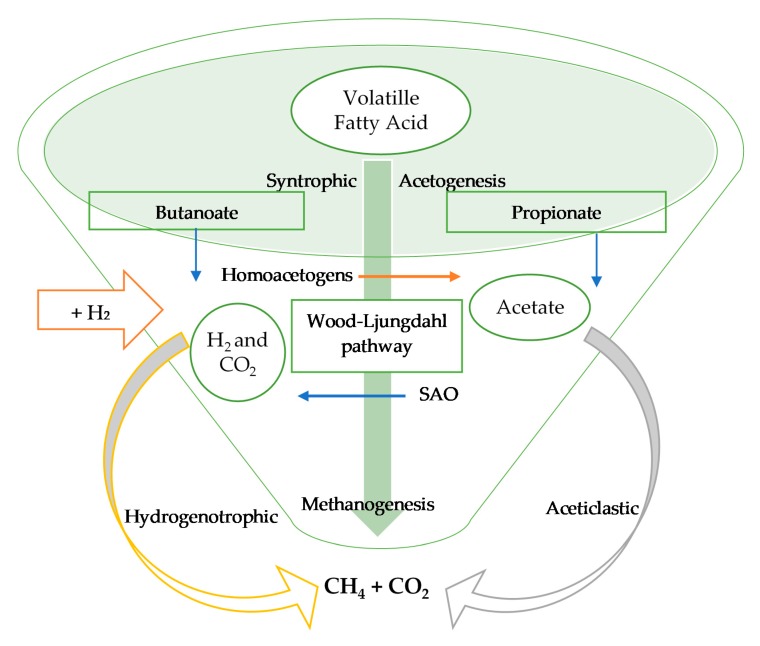
Metabolic pathways for hydrogen assisted methanogenesis [25].

The CO_2_ is converted to acetate acid with the help of homoacetogenic bacteria. Then the acetate acid is converted into CH_4_ with the present of acetoclastic methanogenic archaea. H_2_ plays a crucial role in the whole process of anaerobic digestion. Exogenous addition of H_2_ results in the increase of both hydrogenotrophic methanogens and homoacetogenic species, producing acetate from H_2_ and CO_2_ [75]. The downside of adding H_2_ to the process is the inhibition of syntrophic acetogens which are involved in propionate and butyrate degradation and syntrophic acetate oxidizers (SAO) [76]. It is important to control the concentration of H_2_ to ensure the equilibrium of biochemical reactions. The process is illustrated in Figure 7.

One type of biogas reactor often used in this process is called “continuous stirred tank reactor” (CSTR). The process is heavily connected to the pH level in the reactor. The main challenge is to prevent a pH value above 8.5 because it will lead to methanogenesis inhibition [77,78]. Another challenge arises from the oxidation of the volatile fatty acid (VFA) and alcohols associated with the concentration of the injected hydrogen. To prevent the increasing of the pH level and VFA oxidation, co-digestion with acidic waste [79] and injection of high H_2_ concentrations in reactor [80] were proposed to solve the problems, respectively. Additionally, a ton of research had been done on how to increase the efficiency of the process. A select few of these are listed in Table 3.

### 3.3. Assessment on Feasibility of Biogas Upgrading

In methanation and biological reaction, costs that need to be considered are investment and operational costs, on top of costs associated with H_2_ electrolysis and methanation. Assumptions made were that a large-scale plant for conversion was constructed and that the declining future cost for H_2_ electrolysis was achieved due to the higher market penetration rate.

#### 3.3.1. Cost Estimation

H_2_ electrolysis involves the production of H_2_ and O_2_ from electricity (renewable) and water. There are two techniques that can carry out hydrolysis, the low-temperature process, and the high- temperature process. However, the lack of flexibility of high-temperature electrolysis had impaired the use of it [83]. Thus, a further assumption was made based on the low-temperature process. Based on these assumptions, investment costs obtained were in the range of 656–768 €/kW; the operating costs were about four percent of it; efficiency was 67%; and electricity consumption was 4.1 kWh/m^3^ [84,85]. The cost of water supply is negligible because it was considered less relevant and can be obtained from the methanation reaction.

For the methanation reaction, besides investment and operating cost, there were costs for capturing CO_2_ from biogas and H_2_ storage. Assuming the implementation of the system was at well-established biogas upgrading units, the cost can be neglected. During methanation, heat was released and will be used to capture the CO_2_ from the biogas, resulting in zero cost on heat generation. The water obtained can be used for H_2_ hydrolysis. The storage of H_2_ in steel tanks is a well-established technology and can be put at 27 €/kWh as investment costs [86]. The investment cost for the methanation plant can be assumed in the range of 652–785 €/kW; and the operating costs were about four percent of it [85]. However, for biological process, the technique is still under development and the cost cannot be estimated.

In addition, estimation of producing methanol from biogas was done by Zhang et al. (2017) [87]. In the literature, different analyses are taken to calculate the cost. For a plant scale of 5 × 10^6^ kg/day methanol, the total cost will be in range of USD 827 million to USD 1036 million. For comparison, capital cost for fossil fuel-based methanol was around USD 480 million [88]. From an economical point of view, it can be concluded that industrial exploitation of biogas has a long path ahead of them to be on the same level with current fossil fuel-based processes. For sure, by upgrading biogas by converting CO_2_ to methane and methanol is relevant but is now not a viable short-term benefit when compared to already established technologies.

#### 3.3.2. Advantages and Disadvantages

The created mixture in the form of biomethane has a strong resemblance to natural gas. Thus, the distribution of biomethane can be done from existing gas pipelines. This displays a major advantage, as the infrastructure for transporting the biomethane already exists. In contrast to H_2_, new distribution network is needed if it became the main energy carrier. Second, production of biomethane can help balance the electric grid. For example, renewables energy such as solar and wind are intermittent and not flexible enough. By producing biomethane, it helps to make use of excess electricity produced whenever the demand is low. On the other hand, biomethane can be used as fuel in a power plant when the demand is high and exceeding the limit of produced electricity. Finally, unlike electricity, biomethane is carbon neutral and can be stored efficiently for future use.

One of the drawbacks of the technique is low efficiency. When converting biogas into biomethane using H_2_, the efficiency is only 60%. In addition, if the biomethane produced was to be used to produce electricity, the efficiency drops to 36%. After analyzing the cost, a question is raised: is this technique economically viable? At the moment, the technique is not viable. However, it is likely to be possible in the future when a system with a large share of intermittent renewables are available.

## 4. Novel Technologies in Carbon Dioxide Conversion

In recent years, the development of new technologies has resulted in the production of a useful commodity by the discovery of new converting processes of CO_2_ from waste and atmosphere. These efforts led to the limiting of GHG emissions to the atmosphere of climate-altering pollutants. While CO_2_ has been safely used for enhanced oil and carbon feedstock, there is an increased focus on identifying options for re-use of CO_2_ for other purposes. There were three stages of development in CO_2_ conversion technologies, which can be classified as past, present, and future [65]. In the past, CO_2_ conversion technologies focused on producing urea, methanol, cyclic carbonate, and salicylic acid. Then its focus shifted to the making of CO_2_ based polymers, fuels, and reactions such as methanation and dry reforming. Meanwhile, CO_2_ conversion technologies in the future are predicted to be focusing on production of carboxylic and succinic acid (SA). Thus, this section will be focusing on the possibility of producing SA from CO_2_ components in biogas.

**Figure 8:**
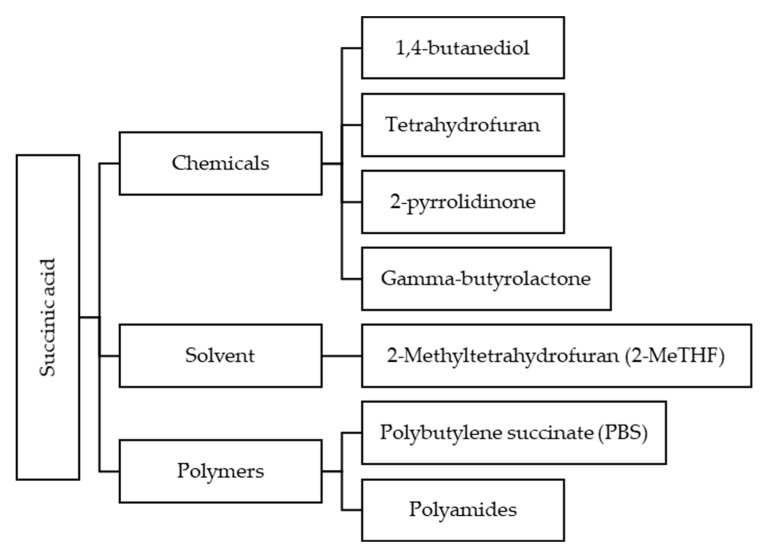
Potential products by using succinic acid (SA) as feedstock [95].

SA (C_4_H_6_O_4_), also known as butanedioic acid is a four-carbon diacid used as a platform for synthesis of various commodities as shown in Figure 8. It is mostly produced from LPG or petroleum oil through specific chemical process. Although, recent analysis revealed that production of bio-SA from bacterial fermentation, which is a renewable source, can be more cost-effective than the traditional processes [89]. In recent years, the advancement of bio-based production of SA was very significant, and as a consequence, a variety of microorganisms has been engineered for the synthesis of SA from sugars, glycerol, or acetate [90]. Furthermore, the CO_2_ is fixed into the bacteria reducing the greenhouse gas emission that lead to pollution. In fact, carbon footprint of bio-SA production is 0.85 kg CO_2_ eq/kg compared to 1.8 kg CO_2_ eq/kg of carbon footprint by petroleum-based SA [91]. One way to operate a CO_2_ fixation process is through reductive tricarboxylic acid (TCA) cycle. In this anaerobic SA production which fully operated under pure CO_2_ condition, 1 mol CO_2_ can produce 1 mol of SA [92]. However, to establish a truly circular bio-economy and utilizing the abundant industrial by-product of CO_2_, valorization of CO_2_ as a substitute to the sugar-based substrates is today of particular relevance [93]. Moreover, if the off-gas from biogas industries could be effectively utilized as a CO_2_ source for SA fermentation, it will simultaneously decrease the cost of the whole process while meeting the commercial-scale requirements for natural gas grid [94].

### 4.1. Simultaneous Biogas Upgrading and Bio-Succinic Acid Production

As mentioned earlier, biogas consists of 60% CH_4_ and 40% CO_2_. The presence of CO_2_ limits the use of biogas. In 2014, Gunnarsson et al. (2014) [96] had come out with a novel approach for converting the CO_2_ component in biogas into SA through a biological process. The study demonstrates a new biogas upgrading technology, which makes use of bacterial fermentation to simultaneously produce high-purity CH_4_ and bio-SA. The microorganism used was a strain of *Actinobacillus succinogenes* 130Z (DSM 22257). Application properties are as follows: Substrate: Glucose 30–32 g/L; reactors: 3-L; T: 37 °C; pH: 6.75; ω: 200 rpm; t: 24 h; P: 101.325 and 140 kPa; gas–liquid ratio: 8.3:1 and 5:1. The results of the study are tabulated in Table 4. Stages of the processes are simplified in Equation (7).

Substrate (*Anaerobic digestion*) → Biogas (60% CH_4_│40% CO_2_) (*Fermentation*) → Natural Gas (95% CH_4_) + Succinate (7)

Based on Table 4, slight over-pressure during fermentation was ideal for the solubility of CO_2_, thus increasing the CH_4_ content in biogas. Increasing the pressure while reducing the ratio also affects other parameters, as CO_2_ consumption rate increased by 16.4%, SA concentration increased by 6.2%, and SA yield increased by 13.8%. The final 95% CH_4_ purity produced was similar to that of commercial biogas upgrading technologies (95–98%) [21]. This study sparks vast potentials for future investigation on the large-scale implementation for practical application in industries. Then in 2018, a group of inspired researchers from Germany, led by Patrick Ballmann, provided a plan to further study this new concept of simultaneous upgrading by replacing the glucose with lignocelluloses from straw [97]. A further modification was done on the straw to provide a suitable strain for SA production while reducing the by-products. To this state, only *A. succinogenes* has been used for SA production coupled with biogas upgrading [25]. That remained the case for a few years until Babaei et al. (2019) [98] conducted an experiment using *Basfia succiniciproducens* (DSM 22022) as a bacterial strain for the fermentation of SA.

The experiment conducted by Babaei et al. (2019) [98] was to determine the possibilities of expanding the simultaneous SA production with a biogas upgrading process by using organic fraction of household kitchen waste (OFHKW) as substrate, replacing the common use of glucose while comparing the performance of *A. succinogenes* and *B. succiniciproducens* in producing SA. OFHKW was broken down by enzymatic hydrolysis to produce monomeric fermentable sugars prior to the fermentation process. The experiment was divided by two major parts: The first was to determine the condition for *B. succiniciproducens* to produce SA, the second was to prove the ability of *B. succiniciproducens* to conduct a simultaneous biogas upgrading with SA production. Application properties, results, and discussion of the study are simplified in Table 5.

This novel approach of using household waste as a substrate to produce SA provides the information on how to accomplish a fermentation process using either *A. succinogenes* or *B. succiniciproducens*. The research will be a benchmark for fellow researchers to utilize other home-grown or local products in the production of SA. Additionally, this study proves the ability of *B. succiniciproducens* to be an alternative as a bacterium capable of converting CO_2_ content in biogas into SA. Nevertheless, further studies still need to be done on other bacteria to identify the possibilities of upgrading biogas while producing SA.

### 4.2. Future Perspective of Succinic Acid Production

These studies proved that both biomethane and biochemical (SA) can be produced by utilizing unconventional biomasses. To further improve the utilization of CO_2_ in biogas, research can be done on metabolic engineering of some other bacteria to produce higher SA titer with no by-products. On top of using *A. succinogenes* 130Z (DSM 22257) and *B. succiniciproducens* (DSM 22022), other bacterial strains had been identified that hold a potential to convert CO_2_ in biogas into SA. Fermentation techniques are also a factor in increasing the SA titer. Some of the bacterial strain and fermentation techniques that can possibly be integrated into SA fermentation technique are listed in Table 6. Although these studies were aimed at the direct conversion of CO_2_ into SA, it will set a base for further research on integrating it in simultaneous biogas upgrading.

**Figure 9:**
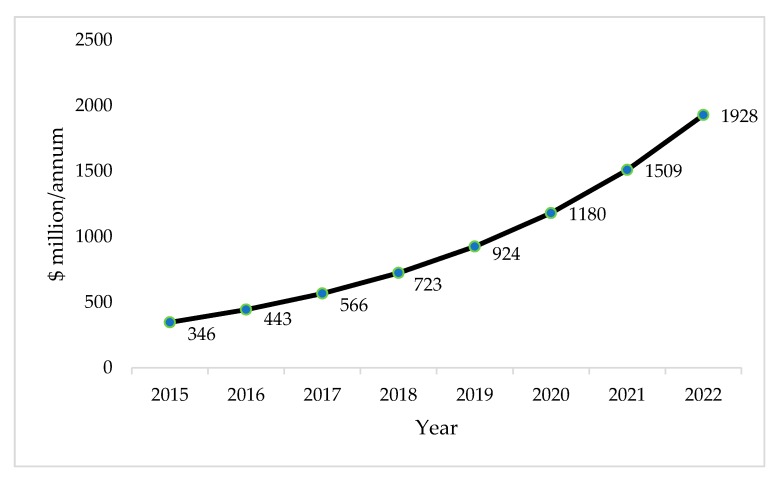
Market forecast of bio-SA volume from the year 2015 to 2022 [112].

Additionally, to implement this technology on a larger scale, further improvement of the simultaneous biogas upgrading, and succinic acid production technology is required. Because there is still no available matured technology in the market, cost breakdown cannot be conducted. Nevertheless, the demand for bio-SA has been increasing over the years. By selling the produced SA, it will reduce the cost of whole operation. Market forecast of bio-SA was conducted by different researchers and can be seen in a simplified form in Figure 9. This reflects the relevance of producing bio-SA in the future.

The evaluation of the performance of microbial conversion of CO_2_ into SA is an important step in providing practical solutions, knowledge, and addressing the gaps in the current understanding [113]. While SA is still widely produced from petrochemical and glucose because of ubiquitous substrate availability, simple process design and high productivities, effort toward producing SA from CO_2_ as sustainable source is still growing and will be applicable if technical barriers that needed to be identified such as limiting gas transfer rates can be overcome [114].

## 5. Conclusions

Global industrial emission of carbon dioxide had risen to an all-time high in 2018 and it is unlikely to reduce soon [115,116]. Growing demand for oil and natural gas globally overshadowed the effort in the development of renewable energy. Furthermore, fossil-fuel infrastructure is still expanding, particularly in developing countries. If current trends continue, the fear of the worst effects of global warming—extreme weather, rising sea levels, plant and animal extinctions, ocean acidification, major shifts in climate, and unprecedented social upheaval—will be inevitable. One of the solutions for these problems is the utilization of bio-natural gas as the substitutes for fossil fuels. In fact, biogas reduces the emission of carbon dioxide while capturing methane, ensuring a cleaner environment. While these are major leaps toward cleaner fuels, still there is room for improvement. Major research had been made from time to time on the techniques to upgrade the biogas to a higher degree. Throughout the years, various technologies and techniques had been developed on how to fully utilize biogas and its by-product so there is no waste release into the environment. One major hurdle for biogas implementation is the cost which hurts its potential employment. While biogas is not the perfect solution for global greenhouse gas emissions, its place in the world of waste management has been very much solidified and will continue to evolve in the coming years.

## Figures and Tables

**Table 1:** Improvement of catalysts in methane production.

Modification	Description/Results	Reference
Ruthenium	More advanced than nickel but costly	[54]
*Electrochemical*		
N-doped carbon	Using the standard three-electrode or H cells Faradaic efficiencies 80% to 94%	[55,56,57]
Copper-on-carbon		
Copper	Electrodeposited on a carbon gas diffusion electrode 38 mA/cm^2^ densities of methane formation	[56]

**Table 2:** Modification of direct hydrogenation of CO_2_ to methanol.

Modification	Description/Result	Reference
Transition metal carbides:		[65]
1. Molybdenum carbide (Mo_2_C) and cementite (Fe_3_C)	High CO_2_ conversion and good methanol selectivity	
2. Tantalum carbide (TaC) and Silicon carbide (SiC)	Almost inactive	
Two-stage bed system	Higher performance	[66]
Heterogeneous copper-based catalysts	Based on CO hydrogenation	[59]
Molybdenum-bismuth bimetallic chalcogenide electrocatalyst	Produce methanol with 70% of Faradaic efficiency with requirement of acetonitrile/ionic liquid electrolyte solution	[67]

**Table 3:** In-situ enriched H_2_ upgrading technologies.

Reactor Type	Upgrading Technology	Substrate	Temperature (°C)	HRT (days)	H_2_ Flow (L/L-days)	pH	CH_4_ (%)	CO_2_ (%)	Reference
1.5 (R1) and 2L (R2) CSTR	a) Mesophilic digester with external H_2_ addition	Cattle ma-nure	35–55	R1 = 25 R2 = 20	R1 = 0.192 R2 = 0.510	R1 = 7.78 R2 = 7.95	89	7	[78]
	b) Thermophilic digester with external H_2_ addition						85	9	
120 mL Batch bottle	Exogenous H_2_ addition	Maize Leaf	52	24	0.04–0.10	7–8	88–89	10–12	[81]
Two 600 mL CSTR	Co-digested substrates with exogenous H_2_ addition	Cattle ma-nure and whey	55	15	1.5–1.7	7.7–7.9	53–75	6.6–13	[79]
Two 3.5 L CSTR	H_2_ addition	Cattle ma-nure	55	14	28.6 mL/L/h	8.3	68	12	[82]

**Table 4:** Performance of the system at different pressure and gas–liquid ratio.

	Pressure (kPa)			
	101.325		140	
Gas-liquid ratio	8.3:1	5:1	8.3:1	5:1
CO_2_ solubility (mM)	9.15	9.15	16.7	16.7
CO_2_ fixation rate (L CO_2_/L-d)	1.35	1.52	2.59	1.77
CH_4_ purity (%)	76.4	85.2	91.1	95.4
SA yield (g/g)	0.60	0.56	0.62	0.63
SA productivity (g/L-h)	0.53	0.53	0.60	0.56
SA concentration (g/L)	12.85	12.74	14.39	13.53
By-products concentration (g/L)	9.5	11.63	8.65	9.96

**Table 5:** Summary of the fermentation process using either *B. succiniciproducens* or *A. succinogenes* as bacterial strain.

Task		Application Properties	Results	Discussion
SA Production		Carbon source: MgCO_3_ 5–100 g/L; Substrate: OFHKW 17, 25, 35 & 60 g/L; Serum bottles: 250-mL; T: 37 °C; pH: 6.7 ± 0.1; ω: 150 rpm	*B. succiniciproducens* SA concentration: Maximum titer of 17.9 ± 0.43 g/L; Overall reaction: Substrate + 2 CO_2_ → 2 lactate + 2 acetate + 2 formate	Higher substrate concentration results in higher SA production; *B. succiniciproducens* is preferred for SA fermentation due to better performances at lower concentration, whereas the by-products were lower
			*A. succinogenes* SA concentration: Maximum titer of 21.1 ± 3.5 g/L	
Simultaneous Upgrading	*B. succiniciproducens*	Carbon source: Biogas; Substrate: OFHKW 17 g/L; Reactors: 3-L; T: 37 °C; pH: 6.7; ω: 200rpm; t: 8 h; P: 130 & 140 kPa	SA concentration: 3.8 ± 0.8 g/L (0.25 g_SA_/g_glucose_); CO_2_ content: 8.0% (v/v) reduction; CH_4_ content: 4.7% (v/v) increase	In term of duration and sugar consumption rate, *B. succiniciproducens* (8 h) is still superior than *A. succinogenes* (24 h); The best way to conduct fermentation process was by gradual additional of substrate instead of starting with high substrate concentration
	*A. succinogenes*	Carbon source: Biogas; Substrate: Glucose 32 g/L; Reactors: 3-L; T: 37 °C; pH: 6.75; ω: 200 rpm; t: 24 h; P: 101.325 & 140 kPa	SA concentration: 14.39 g/L; CH_4_ content: 31% (v/v) increase	

**Table 6:** Summary of performances of succinic acid fermentation studies by various microorganisms.

Microorganism	Reactor Type/ Fermentation Technique	Substrate	Titer (g/L)	Yield (g/g)	Reference
A. *succinogenes*	Repeat-batch	Glucose	33.9	0.86	[99]
A. *succinogenes* 130Z	Suspended cell	Glucose	10.4	0.27–0.73	[99]
A. *succinogenes* 130Z	Recycled cell	Glucose	18.6	0.50–0.59	[100]
A. *succinogenes* 130Z	Batch	Whey	21.5	0.57	[101]
A. *succinogenes* 130Z	Continuous	Corn	39.6	0.78	[102]
A. *succinogenes* FZ53	Batch	Glucose	105.8	0.8	[103]
M. *succiniciproducens*	Batch	Glucose	14	0.7	[104]
M. *succiniciproducens*	Batch	Whey	13.5	0.72	[105]
M. *succiniciproducens* MBEL55E	Suspended cell	Lactose	10.3	0.63–0.69	[105]
M. *succiniciproducens* MBEL55E	Suspended cell	Glucose	14.1	0.34–0.61	[100]
		Xylose			
M. *succiniciproducens* MBEL55E	Recycled cell	Glucose	12.8	0.48–0.64	[100]
M. *succiniciproducens* LPK7	Recycled cell	Glucose	12.9	0.10–0.71	[106]
A. *succiniciproducens*	Continuous	Whey	24	0.72	[103]
A. *succiniciproducens* ATCC No. 29305	Suspended cell	Lactose	24.0	0.62–0.72	[107]
A. *succiniciproducens* ATCC No. 29305	Suspended cell	Lactose	14.0	0.81–0.94	[108]
A. *succiniciproducens* ATCC No. 29305	Suspended cell	Glucose	29.6	0.73–0.82	[109]
A. *succiniciproducens* ATCC No. 29305	Suspended cell	Glycerol	16.1	1.23–1.50	[110]
A. *succiniciproducens* ATCC No. 53488	Recycled cell	Glucose	16.5	0.74–0.83	[111]

## References

[1] Höök M., Tang X. (2013). Depletion of fossil fuels and anthropogenic climate change—A review. Energy Policy.

[2] Goede A., van de Sanden R. (2016). CO_2_-Neutral fuels. Europhys. News.

[3] Ragauskas A.J., Williams C.K., Davison B.H., Britovsek G., Cairney J., Eckert C.A., Frederick W.J.J., Hallett J.P., Leak D.J., Liotta C.L. (2006). The path forward for biofuels and biomaterials. Science.

[4] Masse D.I., Talbot G., Gilbert Y., Caruana D.J., Olsen E.A. (2012). A scientific review of the agronomic, environmental and social benefits of anaerobic digestion. Anaerobic Digestions.

[5] Mata-Alvarez J., Macé S., Llabrés P. (2000). Anaerobic digestion of organic solid wastes: An overview of research achievements and perspectives. Bioresour. Technol..

[6] Muhammad Nasir I., Mohd Ghazi T.I., Omar R. (2012). Production of biogas from solid organic wastes through anaerobic digestion: A review. Appl. Microbiol. Biotechnol..

[7] Carlsson M., Lagerkvist A., Morgan-Sagastume F. (2012). The effects of substrate pre-treatment on anaerobic digestion systems: A review. Waste Manag..

[8] Long J.H., Aziz T.N., Reyes F.L., de los Ducoste J.J. (2012). Anaerobic co-digestion of fat, oil, and grease (FOG): A review of gas production and process limitations. Process Saf. Environ. Prot..

[9] Montalvo S., Guerrero L., Borja R., Sánchez E., Milán Z., Cortés I., de la la Rubia M.A. (2012). Application of natural zeolites in anaerobic digestion processes: A review. Appl. Clay Sci..

[10] Raboni M., Urbini G. (2014). Production and use of biogas in Europe: A survey of current status and perspectives. Ambient. Agua.

[11] Kárászová M., Sedláková Z., Izák P. (2015). Gas permeation processes in biogas upgrading: A short review. Chem. Pap..

[12] Sun Q., Li H., Yan J., Liu L., Yu Z., Yu X. (2015). Selection of appropriate biogas upgrading technology—A review of biogas cleaning, upgrading and utilisation. Renew. Sustain. Energy Rev..

[13] Kougias P.G., Treu L., Benavente D.P., Boe K., Campanaro S., Angelidaki I. (2017). Ex-situ biogas upgrading and enhancement in different reactor systems. Bioresour. Technol..

[14] Abatzoglou N., Boivin S. (2009). A review of biogas purification processes. Biofuels Bioprod. Biorefin..

[15] Ryckebosch E., Drouillon M., Vervaeren H. (2011). Techniques for transformation of biogas to biomethane. Biomass Bioenergy.

[16] Bauer F., Persson T., Hulteberg C., Tamm D. (2013). Biogas upgrading—Technology overview, comparison and perspectives for the future. Biofuels Bioprod Biorefin..

[17] Aziz N.I.H.A., Hanafiah M.M., Gheewala S.H. (2019). A review on life cycle assessment of biogas production: Challenges and future perspectives in Malaysia. Biomass Bioenergy.

[18] García-Gutiérrez P., Jacquemin J., McCrellis C., Dimitriou I., Taylor S.F.R., Hardacre C., Allen R.W.K. (2016). Techno-economic feasibility of selective CO_2_ capture processes from biogas streams using ionic liquids as physical absorbents. Energy Fuels.

[19] Andriani D., Wresta A., Atmaja T.D., Saepudin A. (2014). A review on optimization production and upgrading biogas through CO_2_ removal using various techniques. Appl. Biochem. Biotechnol..

[20] Awe O.W., Zhao Y., Nzihou A., Minh D.P., Lyczko N. (2017). A Review of biogas utilisation, purification and upgrading technologies. Waste Biomass Valoriz..

[21] Leonzio G. (2016). Upgrading of biogas to bio-methane with chemical absorption process: Simulation and environmental impact. J. Clean. Prod..

[22] Xia A., Cheng J., Murphy J.D. (2016). Innovation in biological production and upgrading of methane and hydrogen for use as gaseous transport biofuel. Biotechnol. Adv..

[23] Patterson T., Esteves S., Dinsdale R., Guwy A. (2011). An evaluation of the policy and techno-economic factors affecting the potential for biogas upgrading for transport fuel use in the UK. Energy Policy.

[24] Angelidaki I., Treu L., Tsapekos P., Luo G., Campanaro S., Wenzel H., Kougias P.G. (2018). Biogas upgrading and utilization: Current status and perspectives. Biotechnol. Adv..

[25] Tock L., Gassner M., Maréchal F. (2010). Thermochemical production of liquid fuels from biomass: Thermo-economic modeling, process design and process integration analysis. Biomass Bioenergy.

[26] Zhao Q., Leonhardt E., MacConnell C., Frear C., Chen S. (2010). Purification technologies for biogas generated by anaerobic digestion. Climate Friendly Farming—Final Report.

[27] Hoyer K., Hulteberg C., Svensson M., Jenberg J., NØrregÅrd Ø. (2016). Biogas Upgrading: A Technical Review.

[28] Zhou K., Chaemchuen S., Verpoort F. (2017). Alternative materials in technologies for biogas upgrading via CO_2_ capture. Renew. Sustain. Energy Rev..

[29] Ullah Khan I., Hafiz Dzarfan Othman M., Hashim H., Matsuura T., Ismail A.F., Rezaei-DashtArzhandi M., Wan Azelee I. (2017). Biogas as a renewable energy fuel—A review of biogas upgrading, utilisation and storage. Energy Convers. Manag..

[30] Lasocki J., Kołodziejczyk K., Matuszewska A. (2015). Laboratory-scale investigation of biogas treatment by removal of hydrogen sulfide and carbon dioxide. Polish J. Environ. Stud..

[31] Georgiou D., Petrolekas P.D., Hatzixanthis S., Aivasidis A. (2007). Absorption of carbon dioxide by raw and treated dye-bath effluents. J. Hazard. Mater..

[32] Deng L., Hägg M.B. (2010). Techno-economic evaluation of biogas upgrading process using CO_2_ facilitated transport membrane. Int. J. Greenh. Gas Control.

[33] Ho M.T., Allinson G.W., Wiley D.E. (2008). Reducing the cost of CO_2_ capture from flue gases using pressure swing adsorption. Ind. Eng. Chem. Res..

[34] Augelletti R., Conti M., Annesini M.C. (2017). Pressure swing adsorption for biogas upgrading. A new process configuration for the separation of biomethane and carbon dioxide. J. Clean. Prod..

[35] An H., Feng B., Su S. (2011). CO_2_ capture by electrothermal swing adsorption with activated carbon fibre materials. Int. J. Greenh. Gas Control.

[36] Plaza M.G., García S., Rubiera F., Pis J.J., Pevida C. (2010). Post-combustion CO_2_ capture with a commercial activated carbon: Comparison of different regeneration strategies. Chem. Eng. J..

[37] Petersson A., Wellinger A. (2009). Biogas Upgrading Technologies—Developments and Innovations.

[38] Bauer F., Hulteberg C., Persson T., Tamm D., Granskning B. (2013). Biogas Upgrading—Review of Commercial Technologies.

[39] Harasimowicz M., Orluk P., Zakrzewska-Trznadel G., Chmielewski A.G. (2007). Application of polyimide membranes for biogas purification and enrichment. J. Hazard. Mater..

[40] Munoz R., Meier L., Diaz I., Jeison D. (2015). A review on the state-of-the-art of physical/chemical and biological technologies for biogas upgrading. Rev. Environ. Sci. Biotechnol..

[41] Green D.W., Perry R.H. (2008). Perry’s Chemical Engineers’ Hand Book.

[42] Grande C.A., Blom R. (2014). Cryogenic adsorption of methane and carbon dioxide on zeolites 4A and 13X. Energy Fuels.

[43] Marsh M., Officer C.E., Krich K., Krich K., Augenstein D., Benemann J., Rutledge B., Salour D. (2005). Biomethane from Dairy Waste: A Sourcebook for the Production and Use of Renewable Natural Gas in California.

[44] Cecchi F., Cavinato C. (2015). Anaerobic digestion of bio-waste: A mini-review focusing on territorial and environmental aspects. Waste Manag. Res..

[45] Gao Y., Jiang J., Meng Y., Yan F., Aihemaiti A. (2018). A review of recent developments in hydrogen production via biogas dry reforming. Energy Convers. Manag..

[46] Aresta M. (2010). Carbon Dioxide as Chemical Feedstock.

[47] Williams M. (2006). The merck index: An encyclopedia of chemicals, drugs, and biologicals.

[48] Stangeland K., Kalai D., Li H., Yu Z. (2017). CO_2_ Methanation: The effect of catalysts and reaction conditions. Energy Procedia.

[49] Jürgensen L., Augustine E., Born J., Holm-nielsen J.B. (2015). Bioresource technology dynamic biogas upgrading based on the Sabatier process: Thermodynamic and dynamic process simulation. Bioresource Technol..

[50] U.S. Energy Information Administration (EIA) (2017). International Energy Outlook 2017.

[51] Su X., Xu J., Liang B., Duan H., Hou B., Huang Y. (2016). Catalytic carbon dioxide hydrogenation to methane: A review of recent studies. J. Energy Chem..

[52] Hashemnejad S.M., Parvari M. (2011). Deactivation and regeneration of nickel-based catalysts for steam-methane reforming. Chin. J. Catal..

[53] Mutz B., Gänzler A.M., Nachtegaal M., Müller O., Frahm R., Kleist W., Grunwaldt J.-D. (2017). Surface oxidation of supported ni particles and its impact on the catalytic performance during dynamically operated methanation of CO_2_. Catalysts.

[54] (2019). National Academies of Sciences Engineering and Medicine Gaseous Carbon Waste Streams Utilization. Gaseous Carbon Waste Streams Utilization: Status and Research Needs.

[55] Manthiram K., Beberwyck B.J., Alivisatos A.P. (2014). Enhanced electrochemical methanation of carbon dioxide with a dispersible nanoscale copper catalyst. J. Am. Chem. Soc..

[56] Qiu Y.-L., Zhong H.-X., Zhang T.-T., Xu W.-B., Li X.-F., Zhang H.-M. (2017). Copper electrode fabricated via pulse electrodeposition: Toward high methane selectivity and activity for CO_2_ electroreduction. ACS Catal..

[57] Sun X., Kang X., Zhu Q., Ma J., Yang G., Liu Z., Han B. (2016). Very highly efficient reduction of CO_2_ to CH_4_ using metal-free N-doped carbon electrodes. Chem. Sci..

[58] Álvarez A., Bansode A., Urakawa A., Bavykina A.V., Wezendonk T.A., Makkee M., Gascon J., Kapteijn F. (2017). Challenges in the greener production of formates/formic acid, methanol, and DME by heterogeneously catalyzed CO_2_ hydrogenation processes. Chem. Rev..

[59] Ganesh I. (2014). Conversion of carbon dioxide into methanol—A potential liquid fuel: Fundamental challenges and opportunities (a review). Renew. Sustain. Energy Rev..

[60] Pérez-Fortes M., Schöneberger J.C., Boulamanti A., Tzimas E. (2016). Methanol synthesis using captured CO_2_ as raw material: Techno-economic and environmental assessment. Appl. Energy.

[61] Saeidi S., Aishah N., Amin S., Reza M. (2014). Hydrogenation of CO_2_ to value-added products—A review and potential future developments. Biochem. Pharmacol..

[62] Klankermayer J., Wesselbaum S., Beydoun K., Leitner W. (2016). Selective catalytic synthesis using the combination of carbon dioxide and hydrogen: Catalytic chess at the interface of energy and chemistry. Angew. Chemie Int. Ed..

[63] Wang W.-H., Himeda Y., Muckerman J.T., Manbeck G.F., Fujita E. (2015). CO_2_ Hydrogenation to formate and methanol as an alternative to photo- and electrochemical CO_2_ reduction. Chem. Rev..

[64] Liu Y., Zhang Y., Wang T., Tsubaki N. (2007). Efficient conversion of carbon dioxide to methanol using copper catalyst by a new low-temperature hydrogenation process. Chem. Lett..

[65] Alper E., Yuksel Orhan O. (2017). CO_2_ Utilization: Developments in conversion processes. Petroleum.

[66] Rahimpour M.R. (2008). A two-stage catalyst bed concept for conversion of carbon dioxide into methanol. Fuel Process. Technol..

[67] Sun X., Zhu Q., Kang X., Liu H., Qian Q., Zhang Z., Han B. (2016). Molybdenum–Bismuth bimetallic chalcogenide nanosheets for highly efficient electrocatalytic reduction of carbon dioxide to methanol. Angew. Chemie Int. Ed..

[68] Wang W., Wang S., Ma X., Gong J. (2011). Recent advances in catalytic hydrogenation of carbon dioxide. Chem. Soc. Rev..

[69] Qiao J., Liu Y., Hong F., Zhang J. (2014). A review of catalysts for the electroreduction of carbon dioxide to produce low-carbon fuels. Chem. Soc. Rev..

[70] White J.L., Baruch M.F., Pander J.E., Hu Y., Fortmeyer I.C., Park J.E., Zhang T., Liao K., Gu J., Yan Y. (2015). Light-Driven heterogeneous reduction of carbon dioxide: Photocatalysts and photoelectrodes. Chem. Rev..

[71] Wu M., Zhang W., Ji Y., Yi X., Ma J., Wu H., Jiang M. (2017). Coupled CO_2_ fixation from ethylene oxide off-gas with bio-based succinic acid production by engineered recombinant *Escherichia coli*. Biochem. Eng. J..

[72] Jürgensen L., Ehimen E.A., Born J., Holm-Nielsen J.B. (2014). Utilization of surplus electricity from wind power for dynamic biogas upgrading: Northern Germany case study. Biomass Bioenergy.

[73] Singhal S., Agarwal S., Arora S., Sharma P., Singhal N. (2017). Upgrading techniques for transformation of biogas to bio-CNG: A review. Int. J. Energy Res..

[74] Stams A.J.M., Plugge C.M. (2009). Electron transfer in syntrophic communities of anaerobic bacteria and archaea. Nat. Rev. Microbiol..

[75] Schuchmann K., Müller V. (2014). Autotrophy at the thermodynamic limit of life: A model for energy conservation in acetogenic bacteria. Nat. Rev. Microbiol..

[76] Demirel B., Scherer P. (2008). The roles of acetotrophic and hydrogenotrophic methanogens during anaerobic conversion of biomass to methane: A review. Rev. Environ. Sci. Biotechnol..

[77] Luo G., Angelidaki I. (2012). Integrated biogas upgrading and hydrogen utilization in an anaerobic reactor containing enriched hydrogenotrophic methanogenic culture. Biotechnol. Bioeng..

[78] Bassani I., Kougias P.G., Treu L., Angelidaki I. (2015). Biogas upgrading via hydrogenotrophic methanogenesis in two-stage continuous stirred tank reactors at mesophilic and thermophilic conditions. Environ. Sci. Technol..

[79] Luo G., Angelidaki I. (2013). Co-digestion of manure and whey for in situ biogas upgrading by the addition of H2: Process performance and microbial insights. Appl. Microbiol. Biotechnol..

[80] Batstone D.J., Keller J., Angelidaki I., Kalyuzhnyi S.V., Pavlostathis S.G., Rozzi A., Sanders W.T., Siegrist H., Vavilin V.A. (2002). The IWA anaerobic digestion model No 1 (ADM1). Water Sci. Technol..

[81] Mulat D.G., Mosbæk F., Ward A.J., Polag D., Greule M., Keppler F., Nielsen J.L., Feilberg A. (2017). Exogenous addition of H2 for an in situ biogas upgrading through biological reduction of carbon dioxide into methane. Waste Manag..

[82] Luo G., Johansson S., Boe K., Xie L., Zhou Q., Angelidaki I. (2012). Simultaneous hydrogen utilization and in situ biogas upgrading in an anaerobic reactor. Biotechnol. Bioeng..

[83] Fasihi M., Bogdanov D., Breyer C. (2016). Techno-Economic assessment of power-to-liquids (PtL) fuels production and global trading based on hybrid *pv*-wind power plants. Energy Procedia.

[84] Caldera U. (2018). Role of seawater desalination in the management of an integrated water and 100% renewable energy based power sector in Saudi Arabia. Water.

[85] Fasihi M., Bogdanov D., Breyer C. (2017). Long-Term hydrocarbon trade options for the Maghreb Region and Europe—Renewable energy based synthetic fuels for a net zero emissions world. Sustainability.

[86] Lövenich A., Fasihi M., Graf A., Kasten P., Langenheld A., Meyer K., Peter F., Podewils C. (2018). The Future Cost of Electricity-Based Synthetic Fuels.

[87] Zhang C., Jun K.-W., Gao R., Kwak G., Park H.-G. (2017). Carbon dioxide utilization in a gas-to-methanol process combined with CO_2_/Steam-mixed reforming: Techno-economic analysis. Fuel.

[88] Stokes H.C. (2002). The Economics of Methanol Production.

[89] Mazière A., Prinsen P., García A., Luque R., Len C. (2017). A review of progress in (bio)catalytic routes from/to renewable succinic acid. Biofuels Bioprod. Biorefin..

[90] Valderrama-Gomez M.A., Kreitmayer D., Wolf S., Marin-Sanguino A., Kremling A. (2017). Application of theoretical methods to increase succinate production in engineered strains. Bioprocess Biosyst. Eng..

[91] Adom F., Dunn J.B., Han J., Sather N. (2014). Life-Cycle fossil energy consumption and greenhouse gas emissions of bioderived chemicals and their conventional counterparts. Environ. Sci. Technol..

[92] Cheng K.-K., Zhao X.-B., Zeng J., Zhang J.-A. (2012). Biotechnological production of succinic acid: Current state and perspectives. Biofuels Bioprod. Biorefin..

[93] Mohan S.V., Modestra J.A., Amulya K., Butti S.K., Velvizhi G. (2016). A circular bioeconomy with biobased products from CO_2_ sequestration. Trends Biotechnol..

[94] Cao W., Wang Y., Luo J., Yin J., Xing J., Wan Y. (2018). Effectively converting carbon dioxide into succinic acid under mild pressure with Actinobacillus succinogenes by an integrated fermentation and membrane separation process. Bioresour. Technol..

[95] Cukalovic A., Stevens C. (2008). V Feasibility of production methods for succinic acid derivatives: A marriage of renewable resources and chemical technology. Biofuels Bioprod. Biorefin..

[96] Gunnarsson I.B., Alvarado-Morales M., Angelidaki I. (2014). Utilization of CO_2_ fixating bacterium *Actinobacillus succinogenes* 130Z for simultaneous biogas upgrading and biosuccinic acid production. Environ. Sci. Technol..

[97] Ballmann P., Dröge S., Wilkens M. (2018). Integrated succinic acid production using lignocellulose and carbon dioxide from biogas plants. Chemie Ing. Tech..

[98] Babaei M., Tsapekos P., Alvarado-Morales M., Hosseini M., Ebrahimi S., Niaei A., Angelidaki I. (2019). Valorization of organic waste with simultaneous biogas upgrading for the production of succinic acid. Biochem. Eng. J..

[99] Urbance S.E., Pometto A.L., Dispirito A.A., Denli Y. (2004). Evaluation of succinic acid continuous and repeat-batch biofilm fermentation by *Actinobacillus succinogenes* using plastic composite support bioreactors. Appl. Microbiol. Biotechnol..

[100] Kim D.Y., Yim S.C., Lee P.C., Lee W.G., Lee S.Y., Chang H.N. (2004). Batch and continuous fermentation of succinic acid from wood hydrolysate by *Mannheimia succiniciproducens* MBEL55E. Enzyme Microb. Technol..

[101] Wan C., Li Y., Shahbazi A., Xiu S. (2008). Succinic acid production from cheese whey using *Actinobacillus succinogenes* 130 Z. Appl. Biochem. Biotechnol..

[102] Bradfield M.F.A., Mohagheghi A., Salvachúa D., Smith H., Black B.A., Dowe N., Beckham G.T., Nicol W. (2015). Continuous succinic acid production by *Actinobacillus succinogenes* on xylose-enriched hydrolysate. Biotechnol. Biofuels.

[103] Song H., Lee S.Y. (2006). Production of succinic acid by bacterial fermentation. Enzyme Microb. Technol..

[104] Lee P.C., Lee S.Y., Hong S.H., Chang H.N. (2002). Isolation and characterization of a new succinic acid-producing bacterium, *Mannheimia succiniciproducens* MBEL55E, from bovine rumen. Appl. Microbiol. Biotechnol..

[105] Lee P.C., Lee S.Y., Hong S.H., Chang H.N. (2003). Batch and continuous cultures of *Mannheimia succiniciproducens* MBEL55E for the production of succinic acid from whey and corn steep liquor. Bioprocess Biosyst. Eng..

[106] Jae Oh I., Lee H., Park C., Lee S.Y., Lee J. (2008). Succinic acid production by continuous fermentation process using *Mannheimia succiniciproducens* LPK7. J. Microbiol. Biotechnol..

[107] Samuelov N.S., Datta R., Jain M.K., Zeikus J.G. (1999). Whey fermentation by *Anaerobiospirillum succiniciproducens* for production of a succinate-based animal feed additive. Appl. Environ. Microbiol..

[108] Lee P.C., Lee W.G., Kwon S., Lee S.Y., Chang H.N. (2000). Batch and continuous cultivation of *Anaerobiospirillum succiniciproducens* for the production of succinic acid from whey. Appl. Microbiol. Biotechnol..

[109] Lee P.C., Lee S.Y., Chang H.N. (2009). Kinetic study of organic acid formations and growth of *Anaerobiospirillum succiniciproducens* during continuous cultures. J. Microbiol. Biotechnol..

[110] Lee P.C., Lee S.Y., Chang H.N. (2010). Kinetic study on succinic acid and acetic acid formation during continuous cultures of *Anaerobiospirillum succiniciproducens* grown on glycerol. Bioprocess Biosyst. Eng..

[111] Lee P.-C., Lee S.-Y., Chang H.-N. (2008). Cell recycled culture of succinic acid-producing *Anaerobiospirillum succiniciproducens* using an internal membrane filtration system. J. Microbiol. Biotechnol..

[112] Tan J.P., Jahim J., Harun S., Wu T.Y. (2017). Overview of the Potential of Bio-Succinic Acid Production from Oil Palm Fronds. J. Phys. Sci..

[113] Köhler K.A.K., Rühl J., Blank L.M., Schmid A. (2015). Integration of biocatalyst and process engineering for sustainable and efficient n-butanol production. Eng. Life Sci..

[114] Liebal U.W., Blank L.M., Ebert B.E. (2018). CO_2_ to succinic acid—Estimating the potential of biocatalytic routes. Metab. Eng. Commun..

[115] Figueres C., Le Quéré C., Mahindra A., Bäte O., Whiteman G., Peters G., Guan D. (2018). Emissions are still rising: Ramp up the cuts. Nature.

[116] Le Quéré C., Andrew R.M., Friedlingstein P., Sitch S., Hauck J., Pongratz J., Pickers P., Korsbakken J.I., Peters G.P., Canadell J.G. (2018). Global carbon budget 2018. Earth Syst. Sci. Data.

